# Identification of Potential Therapeutic Targets and Molecular Regulatory Mechanisms for Osteoporosis by Bioinformatics Methods

**DOI:** 10.1155/2021/8851421

**Published:** 2021-03-10

**Authors:** Li Zhang, Yunlong Yang, Dechun Geng, Yonghua Wu

**Affiliations:** ^1^Department of Geriatrics, The Municipal Hospital of Suzhou, Jiangsu, China; ^2^Department of orthopedics, The First Affiliated Hospital of Soochow University, Jiangsu, China

## Abstract

**Background:**

Osteoporosis is characterized by low bone mass, deterioration of bone tissue structure, and susceptibility to fracture. New and more suitable therapeutic targets need to be discovered.

**Methods:**

We collected osteoporosis-related datasets (GSE56815, GSE99624, and GSE63446). The methylation markers were obtained by differential analysis. Degree, DMNC, MCC, and MNC plug-ins were used to screen the important methylation markers in PPI network, then enrichment analysis was performed. ROC curve was used to evaluate the diagnostic effect of osteoporosis. In addition, we evaluated the difference in immune cell infiltration between osteoporotic patients and control by ssGSEA. Finally, differential miRNAs in osteoporosis were used to predict the regulators of key methylation markers.

**Results:**

A total of 2351 differentially expressed genes and 5246 differentially methylated positions were obtained between osteoporotic patients and controls. We identified 19 methylation markers by PPI network. They were mainly involved in biological functions and signaling pathways such as apoptosis and immune inflammation. HIST1H3G, MAP3K5, NOP2, OXA1L, and ZFPM2 with higher AUC values were considered key methylation markers. There were significant differences in immune cell infiltration between osteoporotic patients and controls, especially dendritic cells and natural killer cells. The correlation between MAP3K5 and immune cells was high, and its differential expression was also validated by other two datasets. In addition, NOP2 was predicted to be regulated by differentially expressed hsa-miR-3130-5p.

**Conclusion:**

Our efforts aim to provide new methylation markers as therapeutic targets for osteoporosis to better treat osteoporosis in the future.

## 1. Introduction

Osteoporosis is defined as a systemic skeletal disease characterized by reduced bone density and deterioration of bone tissue microstructure, resulting in increased bone fragility and sensitivity to fracture [1]. The clinical definition of osteoporosis is that the bone mineral density (BMD) measured by dual-energy X-ray absorptiometry (DEXA) is below the average level in young people [2]. In the absence of effective treatment, as many as one in every two Americans over the age of 50 will develop osteoporosis by 2020 [3]. In Italy, approximately 3.5 million women and 1 million men suffer from osteoporosis [4]. The number of fractures worldwide is expected to double in the coming decades and even by 2040 [5]. The high social and personal costs incurred by osteoporosis pose a challenge to public health and physicians, especially because most patients with osteoporosis remain untreated. This has prompted researchers to develop new diagnostic markers and therapeutic targets for osteoporosis in recent years.

Osteoporosis is caused by an imbalance in bone remodeling. To maintain bone homeostasis, the functions of osteoblasts and osteoclasts are coordinated by a variety of molecules [6]. Studies have shown that the spontaneous increase of proinflammatory cytokines such as interleukin- (IL-) 6, tumor necrosis factor alpha, and osteoclasts enhance the ability of osteoclasts to absorb bone, thus promoting the occurrence of osteoporosis [7, 8]. It has also been observed clinically that the degree of osteoporosis corresponds to the degree of inflammation [9]. Many factors interact, and each gene also plays an important role [10, 11].

microRNAs (miRNAs) are a superfamily of small molecules (22 nucleotides), single-stranded noncoding RNAs [12]. Due to the advancement of high-throughput sequencing technology, more and more new miRNAs have been found to be associated with osteoporosis [13, 14]. miRNAs participate in osteoporosis research by regulating target genes [15]. On the other hand, some gene methylation is also associated with osteoporosis [16]. Studies speculate that methylation modification of genes in osteoporotic patients may be a compensatory mechanism to combat osteoporosis-related bone loss [17]. So far, large numbers of differential methylated CPGs associated with BMD have been identified in bone specimens with large bone density differences [18].

However, the pathogenesis of osteoporosis is still complex and has not been fully elucidated. Multiomics data can provide a clearer and more comprehensive understanding of the pathogenesis of osteoporosis. In this study, we attempt to study the molecular mechanism of osteoporosis through sequencing data by using bioinformatics methods. It aims to reveal potential therapeutic targets.

## 2. Materials and Methods

### 2.1. Data Sources

The sequencing datasets were collected from the gene expression omnibus (GEO) database. We screened the osteoporosis related datasets with a sample size greater than 10. GSE56815 included gene expression profiling of circulating monocytes from 40 extremely high and 40 extremely low hip bone mineral density (BMD) subjects. GSE62402 included transcriptome gene expression of peripheral blood monocytes from 5 low BMD to 5 high BMD subjects. GSE13850 included gene expression profiling of B cells was isolated from 10 low BMD to 10 high BMD patients. All the expression data had been preprocessed using RMA (robust multiarray average) normalization. GSE99624 included methylation profiling of whole peripheral blood from 32 primary osteoporotic patients and 16 control individuals. Data were normalized with internal controls according to standard procedures of Illumina. Methylation level at each locus was calculated with the GenomeStudio Methylation module as beta-value (ranging from 0 to 1). GSE63446 included microRNA profiling of 10 samples. miRNA QC Tool software was used for data summarization, normalization, and quality control.

### 2.2. Analysis of Differentially Expressed Genes and Methylation Position

The differentially expressed genes were obtained from high and low BMD subjects through the limma R software package. The differentially methylation positions were obtained by chAMP software package. The *P* value < 0.05 as threshold for nominally significant differential expression. Gene expression and methylation were in opposite directions and were considered methylation markers which regulated by methylation.

### 2.3. Protein-Protein Interaction Network

The protein-protein interaction (PPI) network was constructed by putting methylation markers into the Search Tool for the Retrieval of Interacting Genes (STRING) (https://string-db.org) [19]. Important methylation markers were obtained through Degree, DMNC, MCC, and MNC plug-ins of Cytoscape, respectively.

### 2.4. Enrichment Analysis

The enrichment analysis of gene ontology (GO) functional analysis and Kyoto Encyclopedia of Genes and Genomes (KEGG) pathway analysis was performed for important methylation markers by the clusterProfile R software package [20]. The cellular component (CC), biological process (BP), and molecular function (MF) terms were included in GO analysis. Gene Set Enrichment Analysis (GSEA) of genes in osteoporosis and control was carried out by the clusterProfile R software package. The *P* value < 0.05 was considered to indicate a statistically significant difference.

### 2.5. Infiltration of Immune Cells

The marker gene set for immune cell types was obtained from Bindea et al. [21]. Single-Sample Gene Set Enrichment Analysis (ssGSEA) program was used to quantify the infiltration levels of immune cell types. The ssGSEA applies gene signatures expressed by immune cell populations to individual samples.

### 2.6. Recognition of miRNA Regulation

An integrative retrieval from TargetScan, a miRNA-target interaction (MTI) database, was applied to search for experimentally validated target miRNAs of key methylation markers. The predicted miRNAs were intersected with differentially expressed miRNAs to obtain regulatory factors that regulate key methylation markers.

## 3. Results

### 3.1. Differentially Expressed Genes and Methylation Position in Osteoporosis

**Figure 1:**
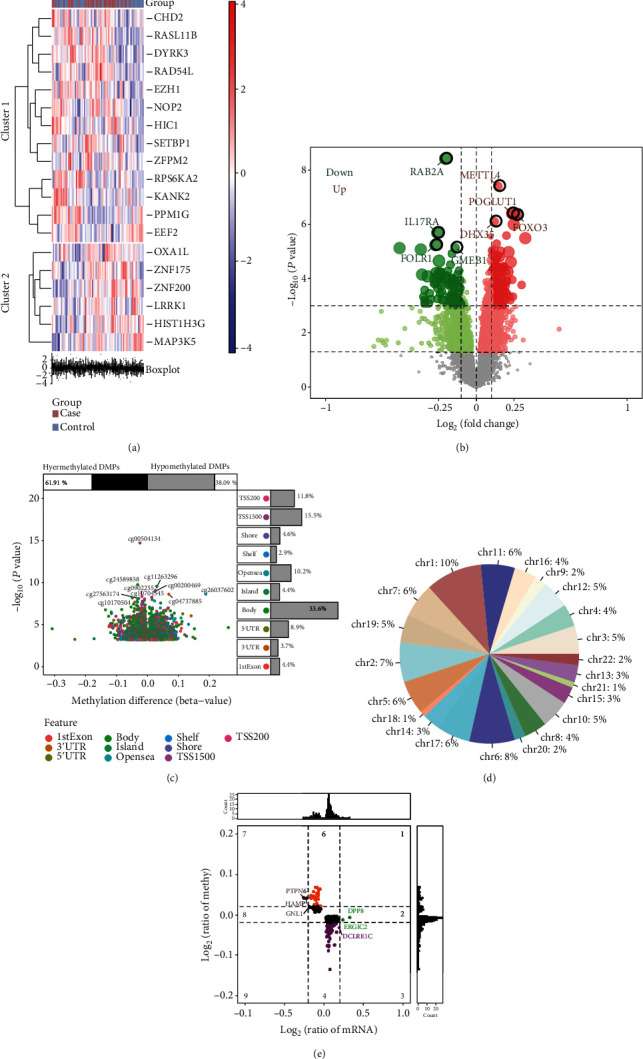
Differentially methylation markers of osteoporosis. (a) Thermogram of differentially expressed genes between osteoporosis and control. (b) The venny map of differentially expressed genes between osteoporosis and control. (c) The difference of methylation position between osteoporosis and control. (d) The proportion of different methylation position in different chromosomes. (e) Expression and methylation level of selected methylation markers.

By comparing the differentially expressed genes (DEGs) between osteoporotic patients and controls, we obtained 2351 statistically significant DEGs (Figure 1(a)). These included 1594 upregulated DEGs and 757 downregulated DEGs (Figure 1(b)). The DEGs may be involved in the disease process of osteoporosis. On the other hand, we compared changes in methylation levels in osteoporotic patients. A total of 5246 differentially methylation positions (DMPs) were found in osteoporotic patients compared with controls (Figure 1(c)). Methylation occurs in a larger proportion at the position of chr1 (Figure 1(d)). Genes whose methylation levels were in opposite directions to those of gene expression levels were considered methylation marks. By comparing DEGs and DMPs, we found 247 genes identified as methylation marks with this property (Figure 1(e)).

### 3.2. Identification of Key Methylation Markers

**Figure 2:**
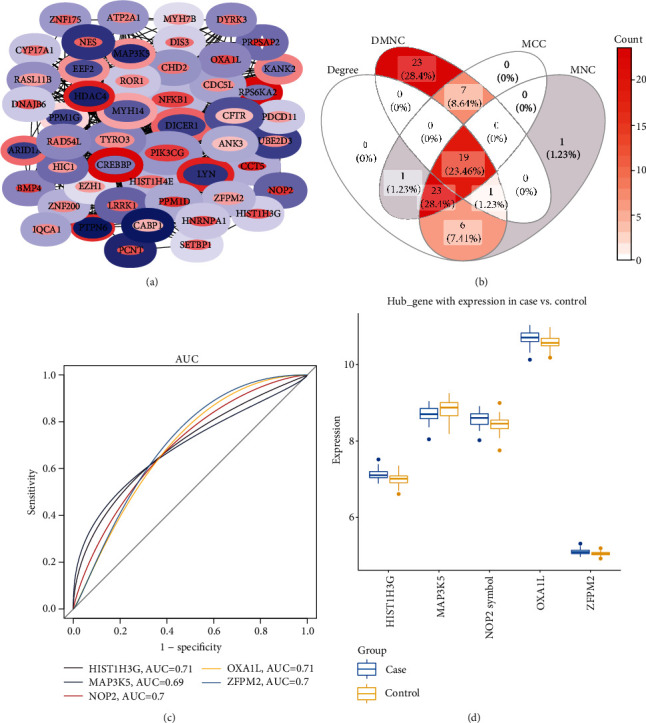
PPI network of methylation markers. (a) The important genes were screened through degree in Cytoscape. The edge of the node was the methylation level, and the nucleus was the expression level. Colors from blue to red indicated that values change from small to large. The size of the node was the degree of the connectivity. (b) The intersection of four important methylation markers. (c) The methylation markers with top 5 AUC values. (d) The differential expression level of key methylation markers.

To further identify key methylation markers, we put 247 genes into the PPI network. Important methylation markers were obtained through Degree, DMNC, MCC, and MNC in Cytoscape, respectively (Figure 2(a), Figure S1). Taking their intersecting genes, we identified 19 methylation markers (Figure 2(b)). Among them, HIST1H3G, MAP3K5, NOP2, OXA1L, and ZFPM2 had AUC values greater than 0.7 were considered key methylation markers (Figure 2(c)). Compared with the control group, HIST1H3G, NOP2, OXA1L, and ZFPM2 were upregulated in osteoporosis, and MAP3K5 was downregulated (Figure 2(d)).

### 3.3. Functional Enrichment of Methylation Markers

**Figure 3:**
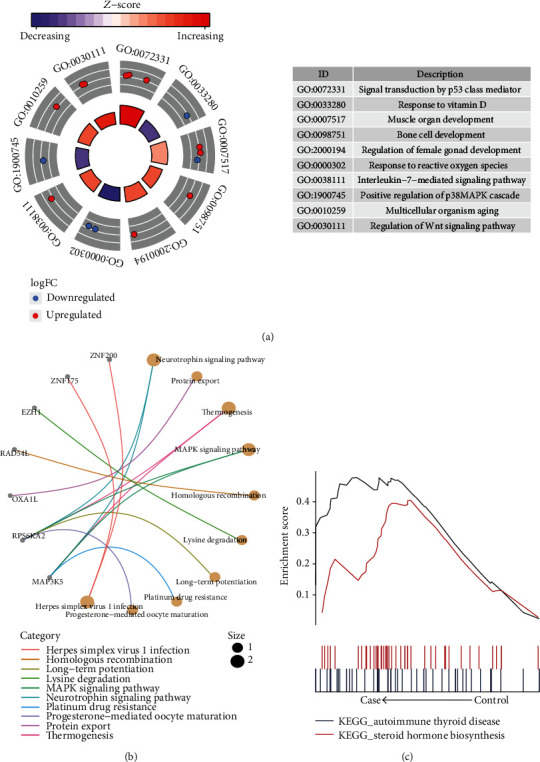
Methylation marker enriched biological functions and signal pathways. (a) The biological process of methylation markers enrichment. (b) The KEGG pathway of methylation markers enrichment. (c) GSEA results of KEGG signaling pathway involving important methylation markers.

Enrichment analysis revealed that 19 methylation markers were significantly involved in biological processes (BP), cell composition (CC), molecular function (MF), and KEGG pathways. It mainly included the upregulated signal transduction by p53 class mediator, and interleukin-7-mediated signaling pathway, and the downregulated response to vitamin D (Figure 3(a)). Enrichment results of KEGG showed that methylation markers were mainly enriched in the MAPK signaling pathway and Neurotrophin signaling pathway (Figure 3(b)). In addition, GSEA results showed that osteoporosis-related methylation markers were significantly enriched in autoimmune thyroid disease, and steroid hormone biosynthesis (Figure 3(c)).

### 3.4. Difference of Immune Infiltration in Osteoporosis

**Figure 4:**
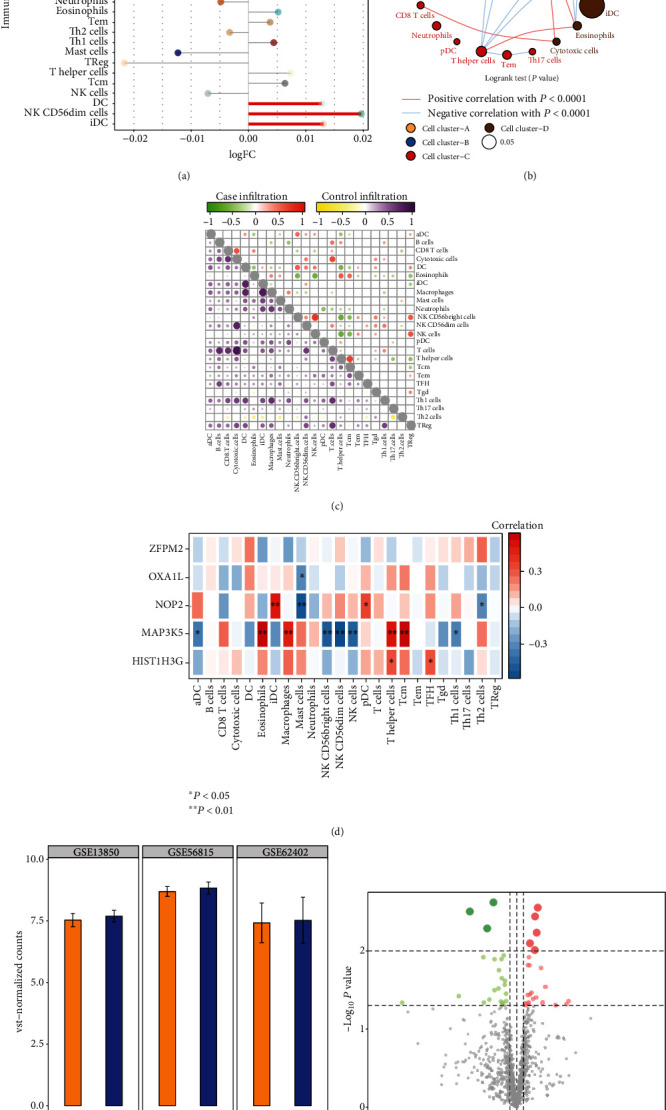
The infiltration of immune cells in osteoporosis. (a) The difference of immune cell infiltration between osteoporosis and control. (b) Clustering of immunoinfiltrating cells. (c) The correlation between immune infiltrating cells in osteoporosis or control samples. (d) The correlation between key methylation markers and immune cells. (e) The differential expression of MAP3K5 in GSE56815, GSE13850, and GSE62402. (f) Differentially expressed miRNAs between osteoporosis and controls.

Compared with the control group, most of the immune cells infiltrated increased in osteoporosis (Figure 4(a)). Among them, iDC, NK CD56dim cells, and DC showed the most significant difference. Differentially infiltrated immune cells were clustered into four categories and positively or negatively correlated with each other (Figure 4(b)). The positive correlation between NK CD56brigh cells and NK cells was the strongest in osteoporosis samples, and that between Cytotoxic cells and T cells was the strongest in control samples (Figure 4(c)). Importantly, among the key methylation markers we identified, MAP3K5 had the strongest correlation with immune cells (Figure 4(d)). The differential expression of MAP3K5 was also validated by GSE13850 and GSE62402. In addition, we obtained differentially expressed miRNAs between osteoporosis and controls, and identified the regulatory relationship between differentially expressed hsa-miR-3130-5p and NOP2 by TargetScan (Figure 4(f)).

## 4. Discussion

In this study, we identified genes modified by methylation through analyzing gene expression and methylation levels in osteoporosis patients. Through the PPI network, we screened the key methylation markers, which were mainly involved in apoptosis, immune inflammation and other related functions. Differences in immune cell infiltration showed that innate immune response was more associated with osteoporosis.

Methylation markers have been acted as biological markers recently [22, 23]. In this study, we also similarly found methylation markers involved in the process of osteoporosis [24]. We identified 19 important methylation markers which enriched some important biological functions and signaling pathways may be associated with osteoporosis. Stability regulation of p53 plays an important role in osteoblast differentiation [25]. At the same time, downregulation of p53 expression may be a potential marker for drug treatment of osteoporosis [26, 27]. Interleukin-7 (IL-7) is a potent osteoclast [28]. IL-7 increases bone loss mainly by increasing T cells produced by RANKL and TNF [29]. Vitamin D and its active metabolites are important components of the immune and hormonal systems, not only controlling phosphorus and calcium homeostasis but also playing an important role in providing a variety of biological effects [30]. Studies have confirmed the importance of maintaining adequate levels of vitamin D to prevent and treat osteoporosis [31]. Interleukin activates priming signals through mitogen-activated protein kinase (MAPK), leading to the expression of proinflammatory cytokines and chemokines, plays a central role in bone resorption, leading to osteoporosis [32]. Drugs can also play a role in the treatment of osteoporosis by inhibiting the activation of the MAPK signaling pathway [33]. Neurotrophin signaling pathway has also been confirmed to be involved in the process of osteoporosis [34].

Among key methylation markers we identified, the MAP3 kinase-5 (MAP3K5), also known as apoptosis signal-regulating kinase 1 (ASK1), is a serine/threonine protein kinase that activates JNK and p38 and induces apoptosis [35]. Importantly, we validated the differential expression of MAP3K5 in three sets of data. Consistent with our findings, NOP2 was identified as a candidate gene associated with bone mineral density (BMD) [36]. OXA1L is differentially expressed in estrogen-exposed osteoblasts and is involved in bone formation [37]. Studies have shown that overexpression of ZFPM 2 inhibits the differentiation of osteoblasts [38]. We obtained better AUC values for key methylation markers, which may have the ability to differentiate osteoporosis. These results once again confirm that methylation modification is associated with the occurrence and development of osteoporosis and that intervening modification of gene methylation may be a potential therapeutic means.

The immune system plays an increasingly important role in bone pathophysiology, which has led to a new research field-bone immunology [39]. From the enrichment results, most methylation markers are involved in the immune inflammatory response. This was also confirmed in the differential results of immune cell infiltration. We found that iDC, NK CD56dim cells, and DC had increased differential infiltration in osteoporosis. Bone remodeling is regulated by the interaction of osteoclasts and osteoblasts with complex factors such as immune cells (DCs, etc.) and cytokines [40]. Bone marrow dendritic cells play an important role in the induction of T cell inflammatory cytokine production, which may be related to postmenopausal bone loss [41]. Invariant natural killer T cells (iNKT) cells have long-term effects on bone physiology in osteoporosis patients and play an important role in bone loss in osteoporosis patients [42]. This suggests that immune changes in osteoporotic patients may be the underlying molecular mechanism of pathogenesis, which may also serve as a potential therapeutic target.

Like other studies, this study has some limitations. Firstly, our analytical data are derived from public database with relatively small sample sizes. Second, important analysis results also require experimental validation of clinical samples.

In conclusion, we conducted a comprehensive bioinformatics analysis and identified a set of target genes potentially relevant for osteoporosis treatment and biological pathways that may lead to changes in bone density. Our results reveal valuable insights into the pathogenesis of osteoporosis and methylation markers that may have a treatment role. These results will help to provide hypotheses for future functional studies.

## Data Availability

The raw data can be accessed from datasets of GSE56815, GSE99624, and GSE63446.

## Supplementary Materials

Supplementary Materials Figure S1. The important genes were screened through DMNC (a), MCC (b), and MNC (c) plug-ins in Cytoscape. The edge of the node was the methylation level and the nucleus were the expression level. Colors from blue to red indicated that values change from small to large. The size of the node was the degree of the connectivity.
