# Supplementary material for: Identification of Potential Therapeutic Targets and Molecular Regulatory Mechanisms for Osteoporosis by Bioinformatics Methods
Source: Biomed Res Int. 2021 Mar 10;2021:8851421. doi: 10.1155/2021/8851421 (PMC7969088; doi:10.1155/2021/8851421)
Supplement: Supplementary Materials — Figure S1. The important genes were screened through DMNC (a), MCC (b), and MNC (c) plug-ins in Cytoscape. The edge of the node was the methylation level and the nucleus were the expression level. Colors from blue to red indicated that values change from small to large. The size of the node was the degree of the connectivity. [file 8851421.f1.docx]

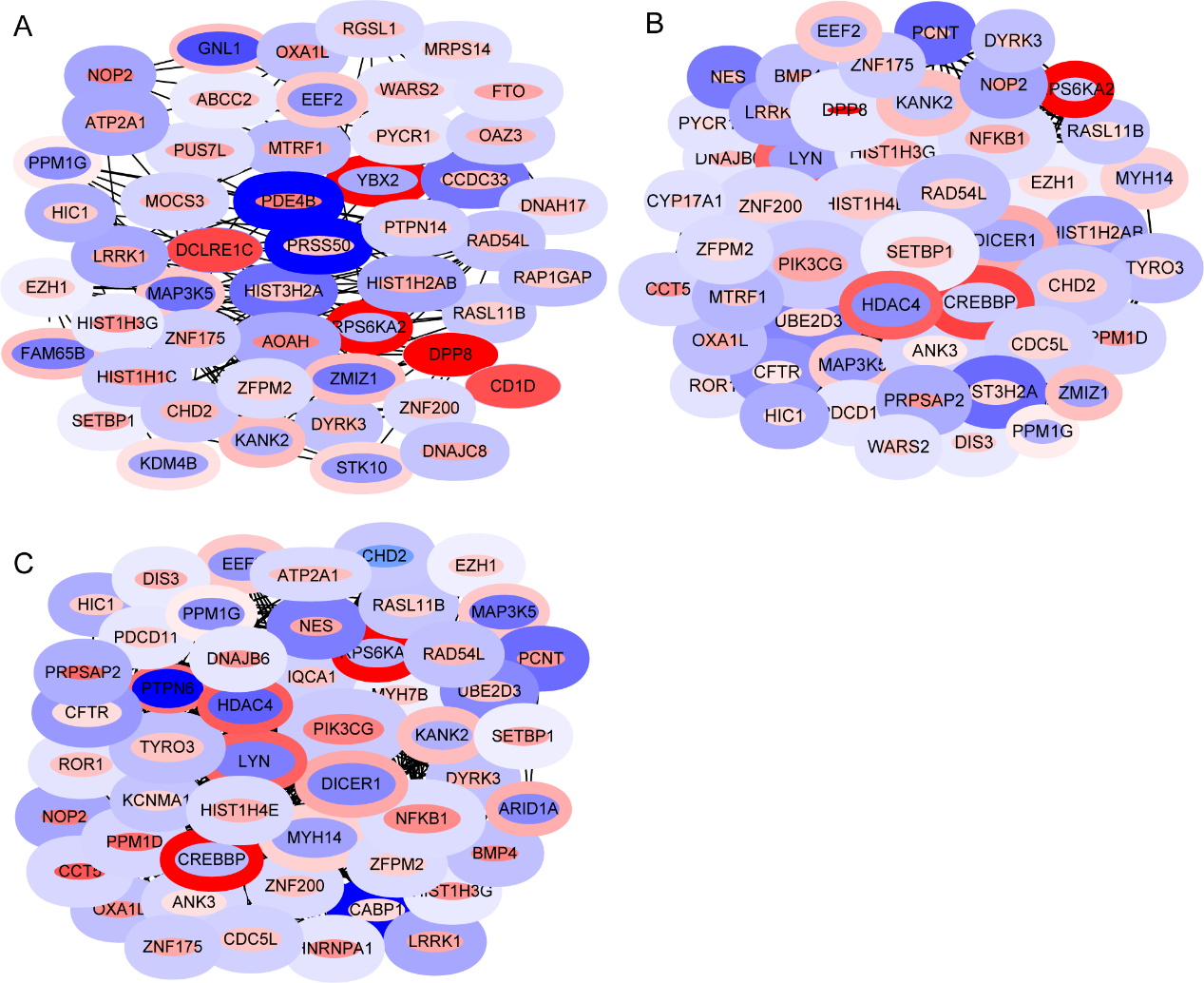


**Figure S1.** The important genes were screened through DMNC (A), MCC (B) and MNC (C) plug-ins in Cytoscape. The edge of the node was the methylation level and the nucleus were the expression level. Colors from blue to red indicated that values change from small to large. The size of the node was the degree of the connectivity.
